# Chloride intracellular channel 3: A secreted pro-invasive oxidoreductase

**DOI:** 10.1080/15384101.2017.1377031

**Published:** 2017-09-29

**Authors:** Jim Norman, Sara Zanivan

**Affiliations:** a Cancer Research UK Beatson Institute, Glasgow, UK; b Institute of Cancer Sciences, University of Glasgow, Glasgow, UK

**Keywords:** angiogenesis, cancer, CAF, CLIC3, integrin, invasion, oxidoreductase, proteomics, stiffness, secretome, TGM2

Tumour progression is accompanied by alterations in the composition and structure of the extracellular matrix (ECM) which creates a microenvironment permissive for cell invasion. The secretome of cancer cells and cancer-associated fibroblasts (CAFs), which are one of the most abundant cell types in the stroma of carcinomas, plays pivotal roles in this process.^1^

Elevated levels of reactive oxygen species (ROS) are often observed in tumours due to increased aerobic metabolism of cancer cells which creates an imbalance between ROS generation and elimination. ROS are intracellular and extracellular signalling molecules that can reversibly modify the thiolate anions of cysteines, and mono-oxidation induced by H_2_O_2_ is an example of such a modification. Cysteine oxidation can cause allosteric changes within the modified protein and this, in turn, can induce alterations to protein function. For this reason, cells have developed a range of strategies to revert protein oxidation. Cytoprotective programmes mediated by thioredoxins and glutaredoxins, which use reduced glutathione (GSH) to restore the reduced status of mono-oxidised cysteines, are notable examples of strategies through which cells attempt to maintain normal protein function.

The human chloride intracellular channel (CLIC) family constitutes a subgroup of the glutathione-S-transferase (GSTs) superfamily and comprises six members, CLIC1 through to CLIC6. CLICs are ∼240 amino acids long and contain a Cysteine-X-X-Cysteine/Serine thioredoxin fold in their N-terminal portion. This fold encompasses a conserved glutaredoxin-like monothiol active site (cysteine 22 in CLIC3). CLIC3 differs from the other members of the family in that its thioredoxin fold contains a dithiol motif with a second cysteine at position 25, which can form an internal disulphide bond with cysteine 22. A number of structural studies have painted a picture of CLICs as metamorphic protein with the capacity to reversibly alternate between a soluble globular conformation comprising a thioredoxin fold (as described above) and a membrane-inserted form consisting predominantly of *β*-sheets. The redox status of the active cysteine may determine which kind of structure CLIC proteins can adopt. CLICs are thought to mediate chloride conductance, hence their name, and it is possible that their ability to bind to and integrate into membranes is linked to this channel/conductance-like property.^2,3^ However, whether CLICs really function as chloride channels and/or mediators of chloride conductance in cells is moot. By contrast, it is now well-established that the CLICs possess enzymatic activity. Al Khamici and co-workers^4^ have clearly demonstrated that CLICs 1, 2 and 4 are glutaredoxin-like enzymes and identified cysteine 24 as the catalytic cysteine residue in CLIC1, which is consistent with its positioning in CLIC1's thioredoxin fold. It is also well-established that CLICs are modulators of intracellular membrane trafficking and endosomal sorting, and enhancers of TGFβ signalling.^2,3^ CLIC3 localises to late endosomes and promotes breast and ovarian cancer cell invasiveness by promoting the delivery of internalised α5β1 integrin and MT1-MMP to the plasma membrane,^5,6^ and CLIC4 also drives trafficking of β1 integrins.^2^

**Figure 1.**
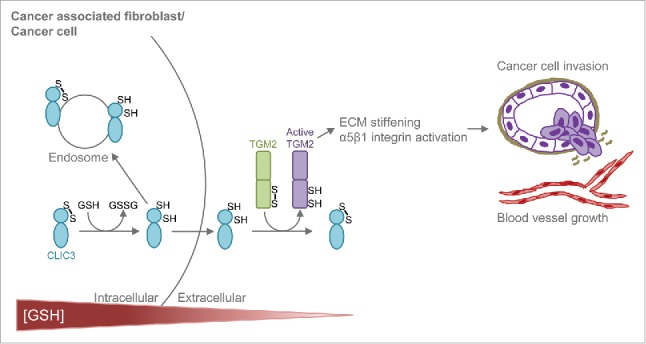
Working model for CLIC3-TGM2. In the reducing intracellular environment CLIC3 is reduced in a GSH-dependent manner and secreted in the oxidative extracellular environment. Extracellularly, CLIC3 reduces (activates) TGM2, which, in turn, stiffens the extracellular matrix (ECM) and activate α5β1 integrin signalling in cancer and endothelial cells, thus inducing tumour invasion and blood vessel growth.

Our recent work on CLIC3 provides key insights into the mechanisms of action of CLICs and their role in cancer.^7^ We have used an unbiased quantitative mass spectrometry (MS)-proteomic approach to determine how the secretome of CAFs drives invasive behaviour of cancer and endothelial cells. Our analysis identified CLIC3 to be a prominent component of the CAF secretome and to be abundant in the stroma of the most aggressive breast and ovarian tumours. We found that soluble secreted CLIC3 possesses GSH-dependent oxidoreductase activity and that this requires its conserved active site cysteine 22. Furthermore, we have identified transglutaminase 2 (TGM2) as a physiological CLIC3 substrate that acts extracellularly to implement CLIC3's effects on cancer invasiveness. Using quantitative MS we have established that CLIC3 can reduce specific cysteines in TGM2. Furthermore we provide evidence that the interaction between CLIC3 and TGM2 controls the binding of TGM2 to its regulatory cofactors in a way that influences its ability to crosslink ECM components to increase tumour stiffness and thus activate pro-invasive *β*1 integrin signalling.. From this we have been able to assemble a picture of how CLIC3 functions in cancer. As fibroblasts become activated in tumours, they express increased levels of CLIC3. This CLIC3 is secreted into the extracellular space whereupon it acts as an oxidoreductase to activate extracellular TGM2. Activated TGM2 then stiffens the ECM and drives integrin dependent invasive processes, such as the disruption of basement membranes by cancer cells and the invasion of endothelial cells into the tumour (Fig. 1). In the context of cancer pathology, our work brings new insights into the mechanism through which CLIC proteins can promote the transition from indolent tumour *in situ* to an aggressive invasive carcinoma.

Our pioneering work on secreted CLIC3 paves the way for a deeper understanding of CLICs' functions as secreted extracellular factors, particularly in the progression of diseases where the levels of these proteins have been found to be altered. Finally, our observation that it is CLIC3's enzymatic activity (and not its ability to function as a chloride conductance) that drives cancer progression, in combination with the fact that this is achieved by CLIC3 acting extracellularly, emphasise how this family of proteins could be targeted pharmacologically. We propose that a search for compounds to selectively oppose the GSH-dependent enzymatic activity of CLIC3 may yield effective therapeutic agents to tackle invasive disease.
